# A Facile Route for the Preparation of Monodisperse Iron nitride at Silica Core/shell Nanostructures

**DOI:** 10.3389/fbioe.2021.735727

**Published:** 2021-09-20

**Authors:** Hoonsub Kim, Pyung Won Im, Yuanzhe Piao

**Affiliations:** ^1^ Graduate School of Convergence Science and Technology, Seoul National University, Suwon, South Korea; ^2^ Department of Neurosurgery, Clinical Research Institute, Seoul National University Hospital, Seoul, South Korea; ^3^ Cancer Research Institute Ischemia/Hypoxia Disease Institute, Seoul National University College of Medicine, Seoul, South Korea; ^4^ Advanced Institutes of Convergence Technology, Suwon, South Korea

**Keywords:** iron oxide, iron nitride, reverse microemulsion, silica, core/shell, nanostructure, MRI

## Abstract

Uniform-sized iron oxide nanoparticles obtained from the solution phase thermal decomposition of the iron-oleate complex were encapsulated inside the silica shell by the reverse microemulsion technique, and then thermal treatment under NH_3_ to transfer the iron oxide to iron nitride. The transmission electron microscopy images distinctly demonstrated that the as-prepared iron nitride at silica core/shell nanostructures were highly uniform in particle-size distribution. By using iron oxide nanoparticles of 6.1, 10.3, 16.2, and 21.8 nm as starting materials, iron nitride nanoparticles with average diameters of 5.6, 9.3, 11.6, and 16.7 nm were produced, respectively. The acid-resistant properties of the iron nitride at silica core/shell nanostructures were found to be much higher than the starting iron oxide at silica. A superconducting quantum interference device was used for the magnetic characterization of the nanostructure. Besides, magnetic resonance imaging (MRI) studies using iron nitride at silica nanocomposites as contrast agents demonstrated *T*_2_ enhanced effects that were dependent on the concentration. These core/shell nanostructures have enormous potential in magnetic nanodevice and biomedical applications. The current process is expected to be easy for large-scale and transfer other metal oxide nanoparticles.

## Introduction

The preparation of monodisperse nanostructured materials is of key importance and has been intensively pursued because they exhibit size- and shape-dependent physical and chemical properties (Goldstein et al., 1992; Xu et al., 1994; Alivisatos, 1996; El-Sayed, 2001; Hyeon, 2003; Daniel and Astruc, 2004; Burda et al., 2005; Im et al., 2005; Zettsu et al., 2006; Schmid, 2010; Muzzio et al., 2019). In recent years, remarkable advances have been made in the synthesis of monodisperse metal oxide nanoparticles (Hyeon et al., 2001; Joo et al., 2003; Seo et al., 2003; Yin and O’Brien, 2003; Park et al., 2004, 2005, 2007; Sun et al., 2004; Tang et al., 2004; Yin et al., 2004, 2005; Liu et al., 2005; An et al., 2006; Kwon et al., 2007; Mendoza-Garcia and Sun, 2016). Furthermore, these monodisperse nanoparticles have been integrated into silica-based nanoparticle systems for catalytical and biomedical applications (Piao et al., 2008a; Joo et al., 2009; Wang et al., 2018; Yang et al., 2019). Of the various nanoparticle systems, iron oxide nanoparticles coated with silica shells have attracted a great deal of interest. On the one hand, silica shell possesses advantages of low cytotoxicity, uniform size, high stability, easy functionalization, and low cost. On the other hand, iron oxide nanoparticle cores provide convenience for removing and recycling the nanoparticle systems by applying an appropriate magnetic field. Truly, silica-coated iron oxide nanoparticles have been diversely functionalized and extensively researched for catalyst recycling and bioseparation (Ge et al., 2008a, 2008b; Panella et al., 2009; Kim et al., 2010; Adams et al., 2018; Cano et al., 2020). However, some industrially important catalytic processes are carried out under corrosive and drastic reaction conditions. It is also reported that some of the endosomes may fuse with lysosomes containing a low-pH medium and appropriate/specific chelates, which dissolve the iron oxide nanoparticles (Arbab et al., 2005). Therefore, the preparation of anticorrosive and magnetically recyclable silica-based monodisperse nanoparticle systems is very important.

Metal nitrides have attracted great attention in recent years due to their interesting properties and a broad range of applications (Niewa and DiSalvo, 1998; Lengauer, 2000; Brese and O’Keeffe, 2005; Miao et al., 2005; Peng et al., 2019). Metal nitrides are characterized by high melting points, hardness, corrosion resistance, and are referred to as refractory hard metals (Sirvio et al., 1982). These physical properties are desired attributes of catalytic materials that require resistance against attrition and sintering under reaction conditions. These materials also demonstrate catalytic advantages over their parent metals in activity, selectivity, and resistance to poisoning. They have been found to be good catalysts for a wide variety of reactions typically catalyzed by noble metals of high cost and limited supply (Ramanathan and Oyama, 1995; Kwon et al., 1999; Li et al., 1999, 2016; Kaskel et al., 2004; Krawiec et al., 2006; Fischer et al., 2008a; Zhang et al., 2016). As a kind of metal nitride, iron nitrides attracted much research interest due to their interesting mechanical, chemical, and magnetic properties (Nakatani et al., 1993; Chatbi et al., 1995; Niederdrenk et al., 1996; Koltypin et al., 1997; Nishimaki et al., 1999; Yamamoto et al., 1999; Tong et al., 2003; Jiang and Gao, 2005; Grafouté et al., 2007; Yu et al., 2017). Iron nitride nanoparticles are a better heat source for magnetic hyperthermia therapy than iron oxide nanoparticles Shibata et al. ( 2019), and they indicate higher saturation magnetization than iron oxide nanoparticles, making them useful for theranostics using magnetic nanoparticles (Namiki et al., 2011). Cytotoxicity also does not show a significant difference with the conventional iron oxide nanoparticles (Shibata et al., 2021).

To date, a number of synthetic procedures have been developed to produce nanostructured metal nitrides, including hydrazide sol-gel synthesis Kim and Kumta (2003), nitridation or ammonolysis of molecular precursors Kaskel et al. (2003), Schwenzer et al. (2005), Choi et al. (2006), solvothermal method Desmoulins-Krawiec et al. (2004), Sardar et al. (2005), Choi and Gillan (2006), various metathesis routes Wang et al. (2002), Joshi et al. (2005) and technique using mesoporous materials as hard templates (Yang and Huang, 2005; Fischer et al., 2007; Fischer et al., 2008b). However, compared with remarkable advances in the synthesis of monodisperse metal oxide nanoparticles Hyeon et al. (2001), Joo et al. (2003), Seo et al. (2003), Yin and O’Brien (2003), Park et al. (2004), Park et al. (2005), Park et al. (2007); Sun et al. (2004), Tang et al. (2004), Yin et al. (2004), Park et al. (2005), Liu et al. (2005), An et al. (2006), Kwon et al. (2007), Mendoza-Garcia and Sun (2016), the methods for the preparation of metal nitrides are rather limited and most of the products are less uniform.

As alternating methods, there are several reports about the transformation of metal oxides into metal nitrides by reacting metal oxides with nitrogen sources (Vaidhyanathan and Rao, 1997; Dezelah IV et al., 2004; Sun and Li, 2004; Luo et al., 2005; Zhao et al., 2005; Zhao et al., 2006; Buha et al., 2007; Chen et al., 2007; Balamurugan et al., 2018). However, these reactions are processed at elevated temperatures, the aggregation and sintering during transformation lead to morphology changes of the final products, thus causing them to lose uniformity.

In a recent study Piao et al. (2008b), we introduced a novel wrap–bake–peel process to induce the transformation of the phases and structures of nanostructured materials while preserving their nanostructural characteristics. In this work, we report the preparation of monodisperse iron nitride at silica core/shell nanostructures by coating silica nanoshell on the uniform-sized iron oxide nanoparticles and thermal treatment under NH_3_ flow to transfer iron oxide to iron nitride. The thermal treatment temperature was investigated to optimize the sample preparation condition. The synthesized iron nitride at silica core/shell nanostructures were characterized by transmission electron microscopy (TEM), X-ray powder diffraction (XRD), and superconducting quantum interference device (SQUID). Magnetic resonance imaging (MRI) using iron nitride at silica nanocomposite as a contrast agent was also studied. The as-prepared iron nitride at silica core/shell nanostructures were highly uniform in particle-size distribution. By using iron oxide nanoparticles of 6.1, 10.3, 16.2, and 21.8 nm as starting materials, iron nitride nanoparticles with average diameters of 5.6, 9.3, 11.6, and 16.7 nm were produced, respectively. The iron nitride at silica core/shell nanostructures showed higher acid-resistant properties as compared to the starting iron oxide at silica. MRI studies using iron nitride at silica nanocomposites as contrast agents demonstrated *T*_2_ enhanced effects which were dependent on the concentration.

## Materials and Methods

### Synthesis of Iron Oxide Nanoparticles

The synthetic scheme is the same as the previously reported procedure (Park et al., 2004). The iron-oleate complex was synthesized by the reaction of iron (III) chloride (FeCl_3_⋅6H_2_O, Aldrich, 98%) and sodium oleate (TCI, 95%). In a typical synthesis of iron oxide nanocrystals, 7.0 g of 1-octadecene (ODE, Aldrich, 90%) was degassed under vacuum at 120°C for 1.5 h, to which 1.26 g of iron-oleate complex (1.40 mmol of Fe) was added at room temperature. The resulting mixture solution was heated at a rate of 3.3°C/min to 320°C and held at this temperature for a given time. The resulting solution containing the nanocrystals was then cooled to room temperature, and 15 ml of ethanol was added to the solution to precipitate the nanocrystals. The nanocrystals were separated by centrifugation and redispersed in hexane.

### Coating Iron Oxide Nanoparticles With Dense Silica Nanoshell

The encapsulation of the iron oxide nanocrystals inside the silica nanoshell was performed by the modification of the reverse microemulsion technique reported elsewhere (Chang et al., 1994; Vestal and Zhang, 2003; Yi et al., 2005; Lee et al., 2006; Kaiser et al., 2017). The iron oxide nanocrystals were mixed with a cyclohexane solution of polyoxyethylene nonylphenyl ether (Igepal CO-520). The formation of silica around the iron oxide nanocrystals was initiated by the successive addition of NH_4_OH aqueous solution and tetraethyl orthosilicate (TEOS). The reaction mixture was kept on stirring for 12 h at room temperature. When acetone was added, solids were precipitated out of the reaction suspension. The resulting solids were isolated by centrifugation and washed several times with ethanol.

### Further Coating With Mesoporous Silica Nanoshell

To further coating of mesoporous silica shell on dense silica-coated iron oxide nanoparticles, a solution containing 2 ml TEOS and 0.8 ml octadecyltrimethoxysilane (C18TMS) was added to a dispersion containing 0.2 g of dense silica-coated iron oxide nanoparticles, 1 ml of ammonium hydroxide (30 wt%), 30 ml of ethanol and 4 ml of deionized water. The mixed solution was reacted for 4 h with vigorous stirring. The resulting octadecyl group incorporated on the silica outer shell/dense silica inner shell/iron oxide core nanocomposite was retrieved by centrifugation, and calcined at 550°C for 6 h under an oxygen atmosphere to produce the final mesoporous silica outer shell/dense silica inner shell/iron oxide core nanostructure.

### Transformation of Iron oxide at silica to Iron nitride at silica Nanostructure

The silica-coated iron oxide nanocomposites were heated at 600°C for 10 h under a flow of 10 sccm NH_3_ to transform iron oxide to iron nitride. Aqueous nanoparticle dispersion was made by immersing the resulting nanocomposite in deionized water with sonication for 5 h.

### Cell Culture

Three types of cells were used in the cytotoxicity test. Two types were cancer cells and the other was normal cells. U87MG (American Type Culture Collection) was one of the glioblastomas, FsaII (department of Tumor Biology, Seoul National University Hospital) was fibrosarcoma, and HFB-141103 (Neurosurgery, Seoul National University Hospital) was the normal form of fibrosarcoma. U87MG and HFB-141103 were cultured in DMEM, 10% FBS, and 1% AA medium and FsaII used deferent media that was RPMI, 10% FBS, 1% AA. All cells were cultured in a 37°C, 5% CO_2_ incubator.

### Cell Viability Assay

CCK-8 assay kit was a common method to measure the cytotoxicity of drugs, but the absorption of the remaining iron nitride at silica nanocomposites disturbed detection. For this reason, we used a mechanical cell counter (aaa, biotech) and Trypan blue to stain live cells. When cells cultured in 100 pie plates reached 80% confluence, they were subcultured in 6-well plates (1 × 105 cells per well). Incubated the plate for 24 h to allow the cells to attach to the plate. After incubation for 24 h, various concentrations (3, 5, 10, 30, 50, 100 μg/ml) of iron nitride at silica nanocomposites were added to each cell and incubated for an additional 24 h. This experiment was replicated three times for accuracy. The relative cell viability (%) was expressed as a percentage relative to the control cells, which were considered 100% viable.

### MR Imaging

MR imaging of the phantoms was performed under a standard head coil and by using a 1.5 T MR imager (Signa Excite; GE Medical Systems, Milwaukee, WI) to obtain the *T*_2_-axial images. The sequence parameters were a repetition time = 4,000 m s, an effective echo time = 123.5 m s, a field of view = 200 × 200 mm, a flip angle = 90°, a matrix = 384 × 320, a slice thickness = 2.0 mm, a slice separation = 2.0 mm and the number of excitations = 2.0. The *T*_2_ values were measured by using the conventional spin-echo (TR/TE = 5,000 m s/16, 20, 32, 40, 48, 50, 60, 64, 80, 100, 150, and 200 m s) with one echo for each sequence while varying the TE. The *T*_2_ values were calculated by fitting the decreased signal intensities with the increasing TEs into a mono-exponential function.

### Characterization

All TEM images were obtained using a JEOL EM-2010 microscope at an acceleration voltage of 200 kV. The samples for TEM studies were prepared by drying a drop of the suspension of the nanoparticles on a piece of the carbon-coated copper grid under ambient conditions. The X-ray diffraction pattern was taken by a Rigaku D/Max-2500 diffractometer system. The particle size was estimated by measuring the sizes of 200 particles from TEM images. Elemental analysis was performed by using inductively coupled plasma atomic emission spectroscopy (ICP-AES, Shimadzu, Japan). Magnetization measurements were performed on a Quantum Design MPMS 5XL SQUID magnetometer. MR imaging of the phantoms was performed under a standard head coil and by using a 1.5 T MR imager (Signa Excite; GE Medical Systems, Milwaukee, WI) to obtain the *T*_2_-axial images. The nitrogen adsorption and desorption isotherms were measured at 77 K using a Micromeritics ASAP 2000 Gas Adsorption Analyzer. Total surface areas and pore volumes were determined using the BET (Brunauer-Emmett-Teller) equation and the single point method, respectively.

## Results and Discussion

**FIGURE 1:**
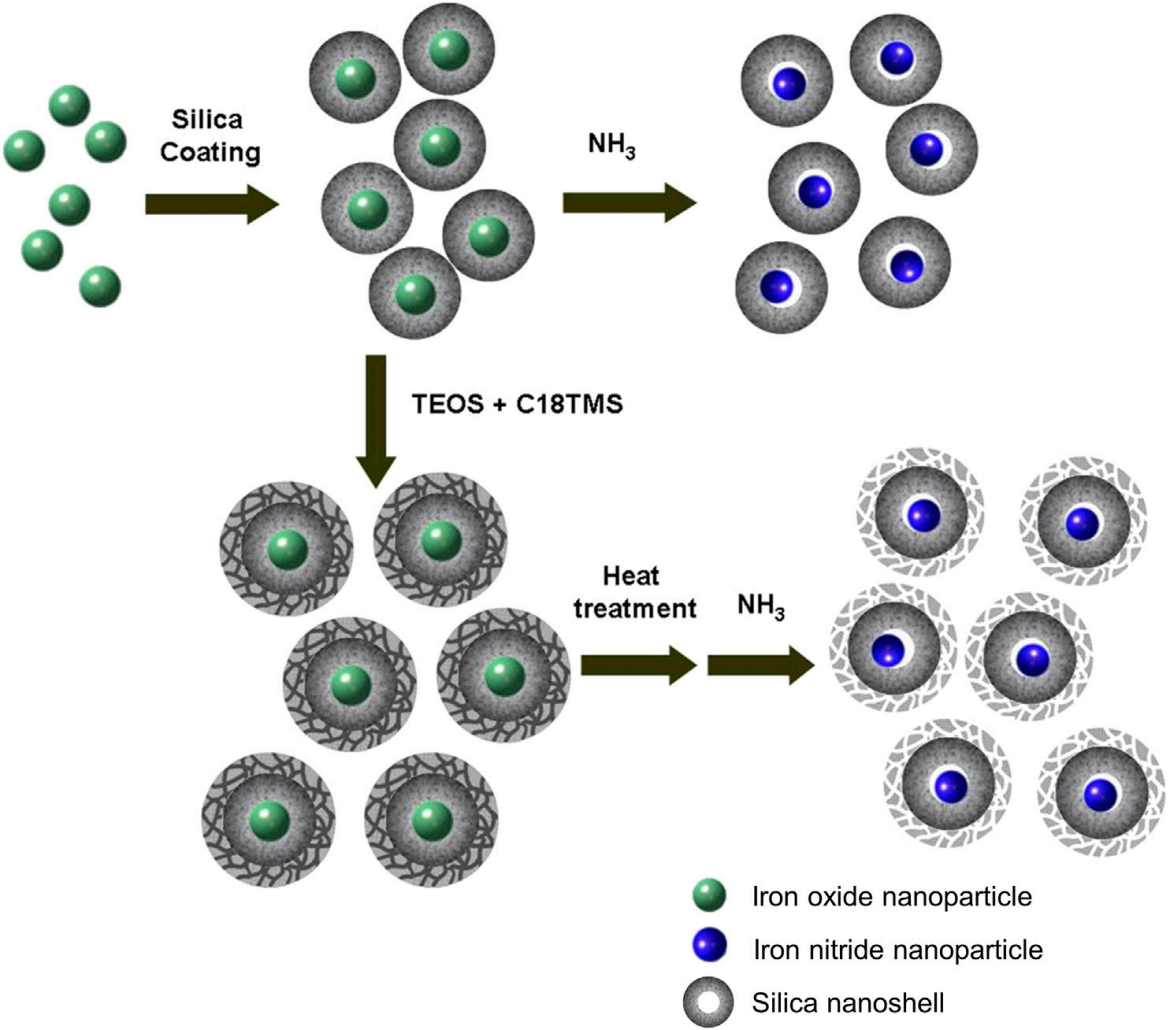
Schematic illustration of the synthetic procedure.

The synthetic process mainly consists of two simple steps as schematically represented in Figure 1, coating iron oxide nanoparticles with silica nanoshell followed by thermal treatment under NH_3_ flux to transfer the iron oxide nanoparticles to iron nitride nanoparticles while the silica shell is served to prevent sintering of the core nanoparticles.

**FIGURE 2:**
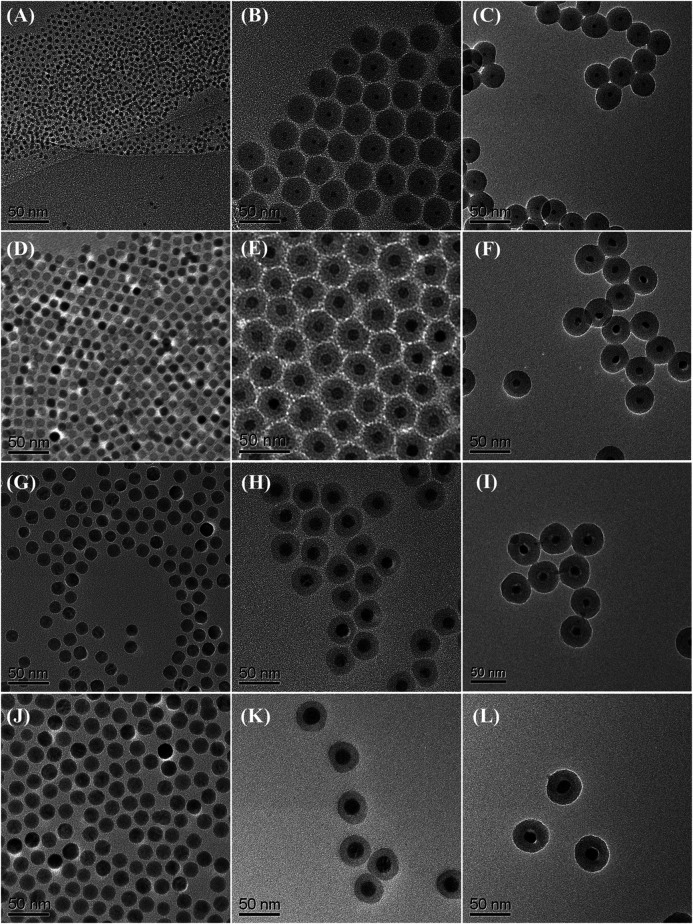
TEM images of starting iron oxide nanoparticles of **(A)** 6.1 nm **(D)** 10.3 nm **(G)** 16.2 nm, and **(J)** 21.8 nm; iron oxide at silica core/shell nanoparticles with **(B)** 6.1 nm **(E)** 10.3 nm **(H)** 16.2 nm, and **(K)** 21.8 nm core **(C)** iron nitride at silica core/shell nanoparticles transferred from **(B) (F)** nanoparticles transferred from **(E) (I)** nanoparticles transferred from **(H)**, and **(L)** nanoparticles transferred from **(K)**.

The preparation starts with the synthesis of highly uniform iron oxide nanoparticles by using a procedure reported previously (Park et al., 2004). Monodispersed iron oxide nanoparticles of 6.1, 10.3, 16.2, and 21.8 nm were used as starting materials (see Figure 2). Since the nanoparticles were hydrophobic, the reverse microemulsion method (Chang et al., 1994; Vestal and Zhang, 2003; Yi et al., 2005; Lee et al., 2006; Kaiser et al., 2017) was used to coat silica nanoshell on each nanoparticle. The thickness of the nanoshell could be well controlled by varying the synthesis and processing parameters. Typical TEM images of the iron oxide nanoparticle at silica nanoparticles with various sized cores are shown in Figures 2B, E, H, K, respectively. These TEM images all reveal uniform iron oxide at silica core/shell nanostructures. The addition of acetone followed by centrifugation enabled the isolation of the silica-coated iron oxide nanoparticles in the form of brown powder. The resulting nanostructures were then put inside a tubular furnace and heated under NH_3_ at 600°C for 10 h. It is obvious from TEM observations that after thermal treatment, although the core nanoparticles were shrunk to the smaller size (from 6.1, 10.3, 16.2, and 21.8 nm of iron oxide to 5.6, 9.3, 11.6, and 16.7 nm of iron nitride, respectively), highly uniform in particle-size distribution was remained and no agglomeration could be observed. (Figures 2C, F, I, L). Large-magnification TEM images of the iron oxide nanoparticles with original diameters of 10.3 nm and the final iron nitride nanoparticles are supplied in the Supplementary Material (Supplementary Figure S3).

**FIGURE 3:**
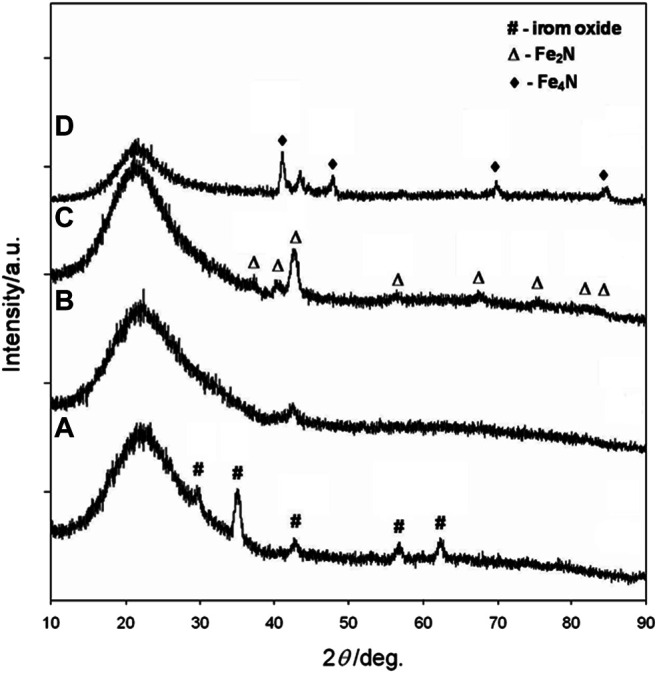
XRD patterns of 10.3 nm iron oxide nanoparticles coated with silica shells and reacted with ammonia gas at **(A)** 400°C **(B)** 500°C **(C)** 600°C, and **(D)** 700°C for 15 h.

In order to study the phase evolution characteristics, the as-prepared silica-coated iron oxide nanocomposite with a core size of 10.3 nm was used as a model precursor material. The precursor was heat-treated at 400°C, 500°C, 600°C, and 700°C, respectively, for 10 h under a flow of 10 sccm NH_3_. In all cases, the heating and cooling rates were 2°C min^−1^ and 5°C min^−1^, respectively. The products were analyzed by X-ray diffraction and the resulting XRD patterns were summarized in Figure 3. The XRD pattern (Figure 3A) obtained after heating the silica-coated iron oxide at 400°C under NH_3_ flow showed no obvious change compared with the precursor (see Supplementary Figure S4). It is therefore clear that the core nanoparticles retained their iron oxide phase after being heated at that temperature under NH_3_ flow. After heat-treatment at 500°C for 10 h, the iron oxide phase was no longer appear and the diffraction peaks for the iron nitride phase appear, indicating that some oxygen atoms were exchanged with nitrogen atoms and the iron nitride phase started to be generated (Figure 3B). A high temperature leads to a high crystallinity of iron nitride. As the heat-treatment temperature was elevated to 600°C, more strong and distinct diffraction peaks were observed (Figure 3C), which correspond to the Fe_2_N patterns (Grafouté et al., 2007). It is worth mention that the core-shell morphology of each sample was remained untouched after the thermal treatment under NH_3_ flow as high as 600°C, (Figures 2C,F,I,L). Further increasing the heat-treatment to 700°C, new diffraction peaks were observed at 41.22°, 47.97°, 70.18 °and 84.76° (Figure 3D), which correspond to the (111) (200) (220), and (311) planes of the Fe_4_N (JCPDS Card No. 83-0875) (Koltypin et al., 1997). However, the narrow XRD peak shape of the Fe_4_N phase pattern hints at the aggregation and enlargement of the particle size. The typical TEM image in Supplementary Material (Supplementary Figure S1) confirmed the destruction of the core-shell structure and aggregation of the core nanoparticles. The above results reflect that the temperature window ranges that the silica nanoshell physically isolates the nanoparticle cores are limited lower than 700°C under NH_3_ flow.

**FIGURE 4:**
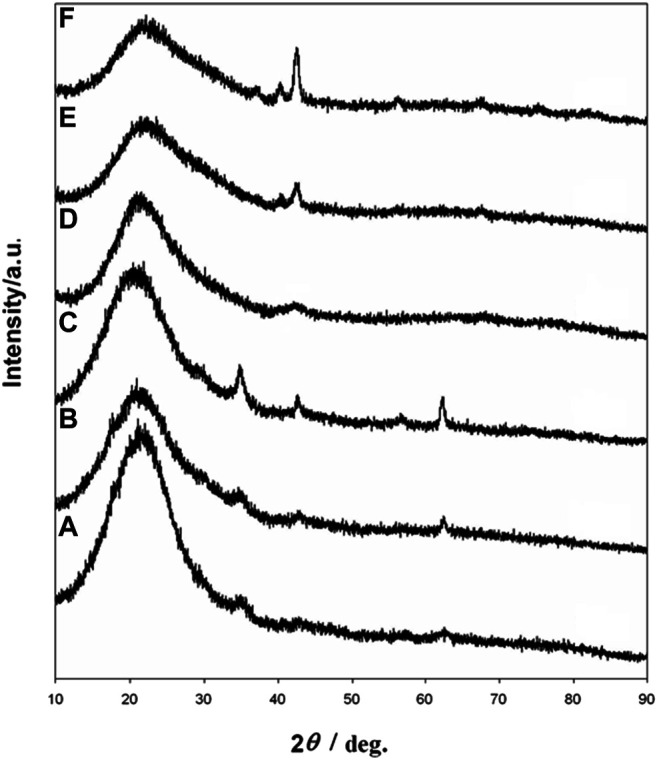
XRD patterns of iron oxide at silica core/shell nanoparticles with **(A)** 6.1 nm **(B)** 16.2 nm, and **(C)** 21.8 nm core **(D)** iron nitride at silica core/shell nanoparticle transferred from **(A) (E)** nanoparticles transferred from **(B) (F)** nanoparticles transferred from **(C)**.

The transformation of iron oxide nanocrystals of different sizes (6.1, 16.2, 21.8 nm) was also confirmed by XRD (Figure 4). According to the results mentioned above, 600°C was chosen as the reaction temperature for the transformation of iron oxide core nanoparticles to iron nitride. When the iron oxide nanocrystals without a silica coating were heated above 500°C under NH_3_ flow, the particles were extensively agglomerated to form micrometer-sized ill-shaped pieces (see Supplementary Material, Supplementary Figure S3). These results show that the silica nanoshell truly serves a critical role in the formation of uniformed iron nitride core nanoparticles in this process.

**FIGURE 5:**
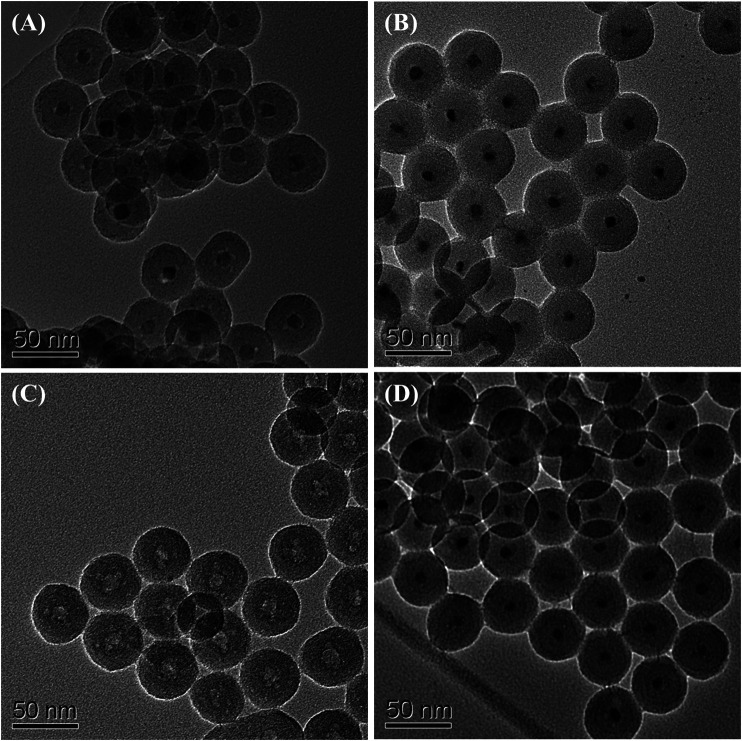
TEM images of iron oxide at silica core/shell nanoparticles after etching with 3 M HCl for **(A)** 2 h, and **(C)** 24 h; iron nitride at silica core/shell nanoparticles after etching with 3 M HCl for **(B)** 2 days, and **(D)** 10 days.

To study the corrosion resistance property, the iron oxide at silica and iron nitride at silica nanoparticles were immersed in HCl, HNO_3_, and H_2_SO_4_, respectively. Iron oxide at silica nanoparticles have only limited acid resistance (Yi et al., 2006) and the dissolution of iron oxide core was observed by the TEM study. After immersing the iron oxide at silica nanoparticles in 3 M HCl for 2 h, the iron oxide cores had been removed partly (Figure 5A). The iron oxide cores were completely removed after etching with 3 M HCl for 24 h and hollow SiO_2_ balls were derived (Figure 5C). In the case of iron nitride at silica nanoparticles, the iron nitride cores were untouched from TEM observation in each acid even after 10 days (Figures 5B, D and Supplementary Material, Supplementary Figure S5, 6).

To understand the acid-resistant properties of the as-prepared iron nitride at silica core/shell nanostructures, we loaded iron oxide nanoparticles on SBA-15 mesoporous silica and further converted them to iron nitride. However, the converted iron nitride nanoparticles were completely dissolved within 24 h by 3 M HCl. Therefore, we speculate that the acid-resistant properties of the as-prepared iron nitride at silica core/shell nanostructures are due to the change of the outer silica coating from a less dense nanoshell to a more compact one during thermal treatment.

Biocompatibility studies of iron nitride at silica core/shell nanostructures were performed in U87MG, FsaII, and HFB-141103 cell lines with different concentrations of iron nitride at silica core/shell nanostructures by CCK-8 analysis. Data obtained after 24 h of cell culture has shown in Supplementary Figure S7. The 3 cell lines have shown a slightly different dependence of viability on the concentration of iron nitride at silica core/shell nanostructures, but cell viability exceeds 70% for all concentrations below 100 μg/ml. High cell viability was maintained. According to the International Organization for Standardization. Part 5: *In vitro* cytotoxicity test for medical devices (ISO 10993-5:2009 guideline), iron nitride at silica nanocomposites at doses less than 100 μg/ml is considered non-cytotoxic against U87MG, FsaII, and HFB-141103 cell lines treated for 24 h.

**FIGURE 6:**
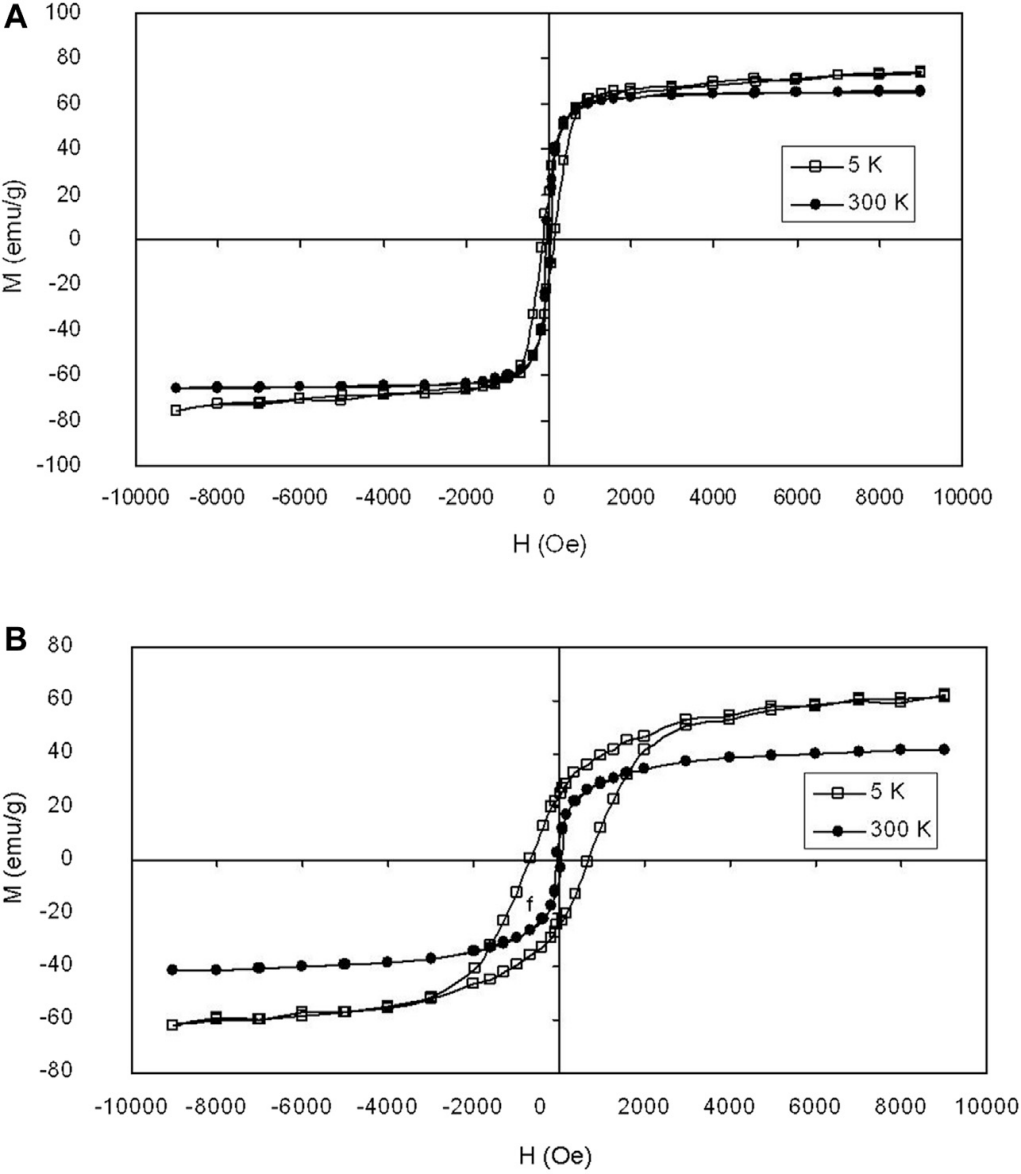
Magnetization loops of **(A)** iron oxide at silica core/shell nanoparticles with 16.2 nm core and **(B)** iron nitride at silica core/shell nanoparticles transferred from **(A)** measured at □) 5 K and •) 300 K, respectively.

Magnetic characterization of the iron oxide at silica and the iron nitride at silica core/shell nanoparticles were performed using a superconducting quantum interference device. The data were presented in emu per g of iron. Figures 6A, B show the typical magnetization curves of the iron oxide at silica and the iron nitride at silica core/shell nanoparticles, respectively, measured by sweeping the external field between −1 and 1 T at □) 5 K and •) 300 K. The magnetization curves of the two nanoparticles all show no remanence or coercivity at room temperature, suggesting the superparamagnetic character. The iron nitride at silica core/shell nanoparticles as described above thus results in a material with good magnetic properties for magnetic separation and recycling as the nanoparticles are not subject to strong magnetic interactions in dispersion. Experimental results show that the water suspension of the iron nitride at silica core-shell nanoparticles only aggregates in a magnetic field.

**FIGURE 7:**
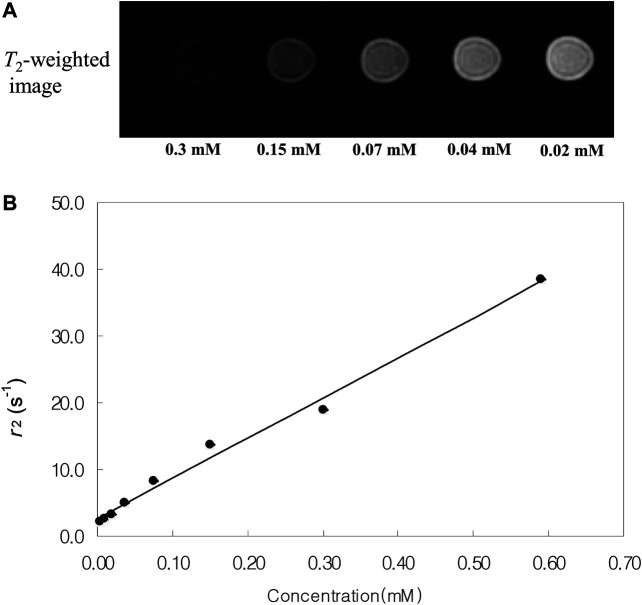
**(A)***T*_2_-weighted MR images and **(B)** spin-spin relaxivity (*r*_2_) of the iron nitride at silica core/shell nanoparticles with 11.6 nm core at various concentrations in water.

Magnetite nanoparticles tend to shorten the spin-spin relaxation times (*T*_2_) of water, resulting in a decrease in the MRI signal intensity. MR imaging of the phantoms was performed under a standard head coil and by using a 1.5 T MR imager to obtain the *T*_2_-axial images. Figure 7 shows *T*_2_-weighted MR images and the plot of the spin-spin relaxivity (*r*_2_) of the iron nitride at silica nanoparticles with 11.6 nm core at various concentrations in water. As the concentration of the iron nitride at silica nanoparticles increases, the signal intensity of the MR image decreases. This behavior allows the iron nitride at silica nanoparticles to be used as a *T*_2_-contrast agent.

**FIGURE 8:**
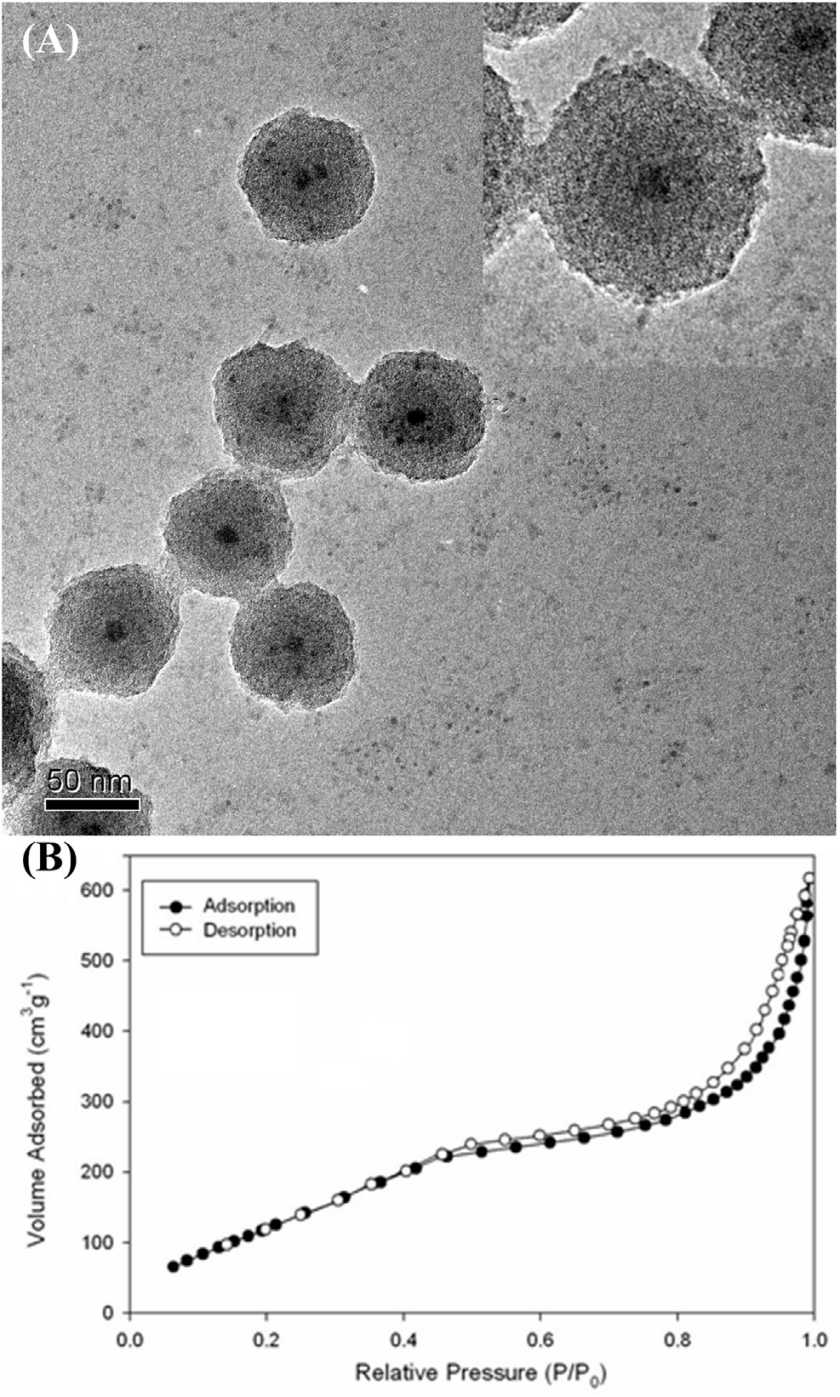
**(A)** TEM image and **(B)** N_2_ adsorption (filled circles) and desorption (empty circles) isotherms of the iron nitride at silica nanoparticles with further coated mesoporous silica outer shell (The inset shows higher-magnification TEM image).

Mesoporous silica nanoshells could be further coated on the core-shell nanoparticles by using a mixture of TEOS and C18TMS as silica sources followed by calcination. Careful inspection of the TEM images shows that the further coated mesoporous nanoshells are rougher than the dense silica inner shell (Figure 8A), indicating the formation of porous structures. Figure 8B shows the N_2_ isotherms of the nanostructured material determined using a Micromeritics ASAP 2000 gas adsorption analyzer. The specific surface area and total pore volume were determined to be 376 m^2^g^−1^, and 0.38 cm^3^ g^-1^, respectively, which are considerably large values as the dense silica-coated solid iron nitride cores have been included in the calculations. It is reported that some drugs could be loaded on the silica mesopore for drug delivery (Zhao et al., 2008; Wen et al., 2017; Manzano and Vallet-Regí, 2020). Therefore, the as-prepared nanostructure has the potential for multifunctional biomedical applications.

## Conclusion

In summary, a facile method was developed for the preparation of monodisperse iron nitride at silica core/shell nanostructures. By using iron oxide nanoparticles of 6.1, 10.3, 16.2, and 21.8 nm as starting materials, iron nitride cores with average diameters of 5.6, 9.3, 11.6, and 16.7 nm were produced, respectively. The iron nitride at silica core/shell nanoparticles showed high acid-resistant properties. Magnetic properties of the nanostructures were studied using a superconducting quantum interference device. Furthermore, MRI studies using iron nitride at silica nanocomposites as contrast agents demonstrated *T*_2_ enhanced effects which were dependent on the concentration. These core/shell nanostructures with well-controlled particle size and morphology have great potential in magnetic nanodevice and biomedical applications. The current process is easily scalable, highly reproducible, and expected to be extended to transfer other metal oxide nanoparticles.

## Data Availability

The original contributions presented in the study are included in the article/Supplementary Material, further inquiries can be directed to the corresponding author.

## References

[B1] Adams S. A. Hauser J. L. Allen A. L. C. Lindquist K. P. Ramirez A. P. Oliver S. (2018). Fe3O4@SiO2 Nanoparticles Functionalized with Gold and Poly(vinylpyrrolidone) for Bio-Separation and Sensing Applications. ACS Appl. Nano Mater. 1, 1406–1412. 10.1021/acsanm.8b00225

[B2] Alivisatos A. P. (1996). Semiconductor Clusters, Nanocrystals, and Quantum Dots. Science 271, 933–937. 10.1126/science.271.5251.933

[B3] An K. Lee N. Park J. Kim S. C. Hwang Y. Park J.-G. (2006). Synthesis, Characterization, and Self-Assembly of Pencil-Shaped CoO Nanorods. J. Am. Chem. Soc. 128, 9753–9760. 10.1021/ja0608702 16866531

[B4] Arbab A. S. Wilson L. B. Ashari P. Jordan E. K. Lewis B. K. Frank J. A. (2005). A Model of Lysosomal Metabolism of Dextran Coated Superparamagnetic Iron Oxide (SPIO) Nanoparticles: Implications for Cellular Magnetic Resonance Imaging. NMR Biomed. 18, 383–389. 10.1002/nbm.970 16013087

[B5] Balamurugan J. Nguyen T. T. Aravindan V. Kim N. H. Lee J. H. (2018). Flexible Solid-State Asymmetric Supercapacitors Based on Nitrogen-Doped Graphene Encapsulated Ternary Metal-Nitrides with Ultralong Cycle Life. Adv. Funct. Mater. 28, 1804663. 10.1002/adfm.201804663

[B6] Brese N. E. O'Keeffe M. (2005). “Crystal Chemistry of Inorganic Nitrides,” in Complexes, Clusters and Crystal Chemistry (Berlin/Heidelberg: Springer-Verlag), 307–378. 10.1007/BFb0036504

[B7] Buha J. Djerdj I. Antonietti M. Niederberger M. (2007). Thermal Transformation of Metal Oxide Nanoparticles into Nanocrystalline Metal Nitrides Using Cyanamide and Urea as Nitrogen Source. Chem. Mater. 19, 3499–3505. 10.1021/cm0701759

[B8] Burda C. Chen X. Narayanan R. El-Sayed M. A. (2005). Chemistry and Properties of Nanocrystals of Different Shapes. Chem. Rev. 105, 1025–1102. 10.1021/cr030063a 15826010

[B9] Cano I. Martin C. Fernandes J. A. Lodge R. W. Dupont J. Casado-Carmona F. A. (2020). Paramagnetic Ionic Liquid-Coated SiO2@Fe3O4 Nanoparticles-The Next Generation of Magnetically Recoverable Nanocatalysts Applied in the Glycolysis of PET. Appl. Catal. B: Environ. 260, 118110. 10.1016/j.apcatb.2019.118110

[B10] Chang S.-Y. Liu L. Asher S. A. (1994). Creation of Templated Complex Topological Morphologies in Colloidal Silica. J. Am. Chem. Soc. 116, 6745–6747. 10.1021/ja00094a033

[B11] Chatbi H. Vergnat M. Bauer P. Marchal G. (1995). Growth and Characterization Studies of Fe4N Thin Films Prepared by Ion Beam Assisted Evaporation. Appl. Phys. Lett. 67, 430–432. 10.1063/1.114622

[B12] Chen H. Nambu A. Wen W. Graciani J. Zhong Z. Hanson J. C. (2007). Reaction of NH3 with Titania: N-Doping of the Oxide and TiN Formation. J. Phys. Chem. C. 111, 1366–1372. 10.1021/jp066137e

[B13] Choi D. Blomgren G. E. Kumta P. N. (2006). Fast and Reversible Surface Redox Reaction in Nanocrystalline Vanadium Nitride Supercapacitors. Adv. Mater. 18, 1178–1182. 10.1002/adma.200502471

[B14] Choi J. Gillan E. G. (2006). Low-temperature Solvothermal Synthesis of Nanocrystalline Indium Nitride and Ga-In-N Composites from the Decomposition of Metal Azides. J. Mater. Chem. 16, 3774–3784. 10.1039/b608204a

[B15] Daniel M.-C. Astruc D. (2004). Gold Nanoparticles: Assembly, Supramolecular Chemistry, Quantum-Size-Related Properties, and Applications toward Biology, Catalysis, and Nanotechnology. Chem. Rev. 104, 293–346. 10.1021/cr030698+ 14719978

[B16] Desmoulins-Krawiec S. Aymonier C. Loppinet-Serani A. Weill F. o. Gorsse S. p. Etourneau J. (2004). Synthesis of Nanostructured Materials in Supercritical Ammonia: Nitrides, Metals and Oxides. J. Mater. Chem. 14, 228–232. 10.1039/b310806f

[B17] Dezelah IV C. L. El-Kadri O. M. Heeg M. J. Winter C. H. (2004). Preparation and Characterization of Molybdenum and Tungsten Nitride Nanoparticles Obtained by Thermolysis of Molecular Precursors. J. Mater. Chem. 14, 3167–3176. 10.1039/b405636a

[B18] El-Sayed M. A. (2001). Some Interesting Properties of Metals Confined in Time and Nanometer Space of Different Shapes. Acc. Chem. Res. 34, 257–264. 10.1021/ar960016n 11308299

[B19] Fischer A. Antonietti M. Thomas A. (2007). Growth Confined by the Nitrogen Source: Synthesis of Pure Metal Nitride Nanoparticles in Mesoporous Graphitic Carbon Nitride. Adv. Mater. 19, 264–267. 10.1002/adma.200602151

[B20] Fischer A. Makowski P. Müller J.-O. Antonietti M. Thomas A. Goettmann F. (2008a). High-Surface-Area TiO2 and TiN as Catalysts for the CC Coupling of Alcohols and Ketones. ChemSusChem. 1, 444–449. 10.1002/cssc.200800019 18702140

[B21] Fischer A. Müller J. O. Antonietti M. Thomas A. (2008b). Synthesis of Ternary Metal Nitride Nanoparticles Using Mesoporous Carbon Nitride as Reactive Template. ACS Nano 2, 2489–2496. 10.1021/nn800503a 19206283

[B22] Ge J. Huynh T. Hu Y. Yin Y. (2008a). Hierarchical Magnetite/silica Nanoassemblies as Magnetically Recoverable Catalyst-Supports. Nano Lett. 8, 931–934. 10.1021/nl080020f 18237148

[B23] Ge J. Zhang Q. Zhang T. Yin Y. (2008b). Core-satellite Nanocomposite Catalysts Protected by a Porous Silica Shell: Controllable Reactivity, High Stability, and Magnetic Recyclability. Angew. Chem. Int. Ed. 47, 8924–8928. 10.1002/anie.200803968 18925600

[B24] Goldstein A. N. Echer C. M. Alivisatos A. P. (1992). Melting in Semiconductor Nanocrystals. Science 256, 1425–1427. 10.1126/science.256.5062.1425 17791609

[B25] Grafouté M. Petitjean C. Rousselot C. Pierson J. F. Grenèche J. M. (2007). Structural Properties of Iron Oxynitride Films Obtained by Reactive Magnetron Sputtering. J. Phys. Condens. Matter 19, 226207. 10.1088/0953-8984/19/22/226207

[B26] Hyeon T. (2003). Chemical Synthesis of Magnetic Nanoparticles. Chem. Commun. 3, 927–934. 10.1039/b207789b 12744306

[B27] Hyeon T. Lee S. S. Park J. Chung Y. Na H. B. (2001). Synthesis of Highly Crystalline and Monodisperse Maghemite Nanocrystallites without a Size-Selection Process. J. Am. Chem. Soc. 123, 12798–12801. 10.1021/ja016812s 11749537

[B28] Im S. H. Lee Y. T. Wiley B. Xia Y. (2005). Large-scale Synthesis of Silver Nanocubes: The Role of HCl in Promoting Cube Perfection and Monodispersity. Angew. Chem. Int. Ed. 44, 2154–2157. 10.1002/anie.200462208 15739241

[B29] Jiang L. Gao L. (2005). Carbon Nanotubes-Metal Nitride Composites: A New Class of Nanocomposites with Enhanced Electrical Properties. J. Mater. Chem. 15, 260–266. 10.1039/b409682g

[B30] Joo J. Yu T. Kim Y. W. Park H. M. Wu F. Zhang J. Z. (2003). Multigram Scale Synthesis and Characterization of Monodisperse Tetragonal Zirconia Nanocrystals. J. Am. Chem. Soc. 125, 6553–6557. 10.1021/ja034258b 12785795

[B31] Joo S. H. Park J. Y. Tsung C.-K. Yamada Y. Yang P. Somorjai G. A. (2009). Thermally Stable Pt/mesoporous Silica Core-Shell Nanocatalysts for High-Temperature Reactions. Nat. Mater 8, 126–131. 10.1038/nmat2329 19029893

[B32] Joshi U. A. Chung S. H. Lee J. S. (2005). Low-temperature, Solvent-free Solid-State Synthesis of Single-Crystalline Titanium Nitride Nanorods with Different Aspect Ratios. J. Solid State. Chem. 178, 755–760. 10.1016/j.jssc.2004.12.032

[B33] Kaiser M. R. Ma Z. Wang X. Han F. Gao T. Fan X. (2017). Reverse Microemulsion Synthesis of Sulfur/Graphene Composite for Lithium/Sulfur Batteries. ACS Nano 11, 9048–9056. 10.1021/acsnano.7b03591 28850776

[B34] Kaskel S. Schlichte K. Chaplais G. Khanna M. (2003). Synthesis and Characterisation of Titanium Nitride Based Nanoparticles. J. Mater. Chem. 13, 1496–1499. 10.1039/b209685d

[B35] Kaskel S. Schlichte K. Kratzke T. (2004). Catalytic Properties of High Surface Area Titanium Nitride Materials. J. Mol. Catal. A: Chem. 208, 291–298. 10.1016/S1381-1169(03)00545-4

[B36] Kim I.-s. Kumta P. N. (2003). Hydrazide Sol-Gel Synthesis of Nanostructured Titanium Nitride: Precursor Chemistry and Phase Evolution. J. Mater. Chem. 13, 2028–2035. 10.1039/b301964k

[B37] Kim J. Piao Y. Lee N. Park Y. I. Lee I.-H. Lee J.-H. (2010). Magnetic Nanocomposite Spheres Decorated with NiO Nanoparticles for a Magnetically Recyclable Protein Separation System. Adv. Mater. 22, 57–60. 10.1002/adma.200901858 20217697

[B38] Koltypin Y. Cao X. Prozorov R. Balogh J. Kaptas D. Gedanken A. (1997). Sonochemical Synthesis of Iron Nitride Nanoparticles. J. Mater. Chem. 7, 2453–2456. 10.1039/a704008c

[B39] Krawiec P. De Cola P. L. Gläser R. Weitkamp J. Weidenthaler C. Kaskel S. (2006). Oxide Foams for the Synthesis of High-Surface-Area Vanadium Nitride Catalysts. Adv. Mater. 18, 505–508. 10.1002/adma.200500278

[B40] Kwon H. Choi S. Thompson L. T. (1999). Vanadium Nitride Catalysts: Synthesis and Evaluation Forn-Butane Dehydrogenation. J. Catal. 184, 236–246. 10.1006/jcat.1999.2450

[B41] Kwon S. G. Piao Y. Park J. Angappane S. Jo Y. Hwang N.-M. (2007). Kinetics of Monodisperse Iron Oxide Nanocrystal Formation by "Heating-Up" Process. J. Am. Chem. Soc. 129, 12571–12584. 10.1021/ja074633q 17887758

[B42] Lee D. C. Mikulec F. V. Pelaez J. M. Koo B. Korgel B. A. (2006). Synthesis and Magnetic Properties of Silica-Coated FePt Nanocrystals. J. Phys. Chem. B. 110, 11160–11166. 10.1021/jp060974z 16771378

[B43] Lengauer W. (2000). “Handbook of Ceramic Hard Materials,” in Handbook of Ceramic Hard Materials. Editor Riedel R. (Weinheim: Wiley VCH). 10.1002/9783527618217

[B44] Li S. Sung Lee J. Hyeon T. Suslick K. S. (1999). Catalytic Hydrodenitrogenation of Indole over Molybdenum Nitride and Carbides with Different Structures. Appl. Catal. A: Gen. 184, 1–9. 10.1016/S0926-860X(99)00044-7

[B45] Li S. Wang Y. Peng S. Zhang L. Al-Enizi A. M. Zhang H. (2016). Co-Ni-Based Nanotubes/Nanosheets as Efficient Water Splitting Electrocatalysts. Adv. Energ. Mater. 6, 1501661. 10.1002/aenm.201501661

[B46] Liu Q. Lu W. Ma A. Tang J. Lin J. Fang J. (2005). Study of Quasi-Monodisperse In2O3Nanocrystals: Synthesis and Optical Determination. J. Am. Chem. Soc. 127, 5276–5277. 10.1021/ja042550t.s003 15826138

[B47] Luo S. Zhou W. Zhang Z. Liu L. Dou X. Wang J. (2005). Synthesis of LongIndium Nitride Nanowires with Uniform Diameters in Large Quantities. Small 1, 1004–1009. 10.1002/smll.200500053 17193386

[B48] Manzano M. Vallet‐Regí M. (2020). Mesoporous Silica Nanoparticles for Drug Delivery. Adv. Funct. Mater. 30, 1902634–1902635. 10.1002/adfm.201902634

[B49] Mendoza-Garcia A. Sun S. (2016). Recent Advances in the High-Temperature Chemical Synthesis of Magnetic Nanoparticles. Adv. Funct. Mater. 26, 3809–3817. 10.1002/adfm.201504172

[B50] Miao M. S. Lukashev P. Herwadkar A. Lambrecht W. R. L. (2005). Crystal Structure, Electronic Structure and Magnetism of Transition Metal Nitrides. Phys. Stat. Sol. (C) 2, 2516–2519. 10.1002/pssc.200461318

[B51] Muzzio M. Li J. Yin Z. Delahunty I. M. Xie J. Sun S. (2019). Monodisperse Nanoparticles for Catalysis and Nanomedicine. Nanoscale 11, 18946–18967. 10.1039/c9nr06080d 31454005

[B52] Nakatani I. Hijikata M. Ozawa K. (1993). Iron-nitride Magnetic Fluids Prepared by Vapor-Liquid Reaction and Their Magnetic Properties. J. Magnetism Magn. Mater. 122, 10–14. 10.1016/0304-8853(93)91028-6

[B53] Namiki Y. Fuchigami T. Tada N. Kawamura R. Matsunuma S. Kitamoto Y. (2011). Nanomedicine for Cancer: Lipid-Based Nanostructures for Drug Delivery and Monitoring. Acc. Chem. Res. 44, 1080–1093. 10.1021/ar200011r 21786832

[B54] Niederdrenk M. Schaaf P. Lieb K.-P. Schulte O. P. Gottingen U. (1996). Characterization of Magnetron-Sputtered ε Iron-Nitride Films. J. Alloys Compounds 237, 81–88. 10.1016/0925-8388(95)02195-7 Institut

[B55] Niewa R. DiSalvo F. J. (1998). Recent Developments in Nitride Chemistry. Chem. Mater. 10, 2733–2752. 10.1021/cm980137c

[B56] Nishimaki K. Ohmae S. Yamamoto T. A. Katsura M. (1999). Formation of Iron-Nitrides by the Reaction of Iron Nanoparticles with a Stream of Ammonia. Nanostructured Mater. 12, 527–530. 10.1016/S0965-9773(99)00175-0

[B57] Panella B. Vargas A. Baiker A. (2009). Magnetically Separable Pt Catalyst for Asymmetric Hydrogenation. J. Catal. 261, 88–93. 10.1016/j.jcat.2008.11.002

[B58] Park J. An K. Hwang Y. Park J.-G. Noh H.-J. Kim J.-Y. (2004). Ultra-large-scale Syntheses of Monodisperse Nanocrystals. Nat. Mater 3, 891–895. 10.1038/nmat1251 15568032

[B59] Park J. Joo J. Kwon S. G. Jang Y. Hyeon T. (2007). Synthesis of Monodisperse Spherical Nanocrystals. Angew. Chem. Int. Ed. 46, 4630–4660. 10.1002/anie.200603148 17525914

[B60] Park J. Lee E. Hwang N.-M. Kang M. Kim S. C. Hwang Y. (2005). One-Nanometer-Scale Size-Controlled Synthesis of Monodisperse Magnetic Iron Oxide Nanoparticles. Angew. Chem. 117, 2932–2937. 10.1002/ange.200461665 15798989

[B61] Peng X. Pi C. Zhang X. Li S. Huo K. Chu P. K. (2019). Recent Progress of Transition Metal Nitrides for Efficient Electrocatalytic Water Splitting. Sustainable Energ. Fuels 3, 366–381. 10.1039/c8se00525g

[B62] Piao Y. Burns A. Kim J. Wiesner U. Hyeon T. (2008a). Designed Fabrication of Silica-Based Nanostructured Particle Systems for Nanomedicine Applications. Adv. Funct. Mater. 18, 3745–3758. 10.1002/adfm.200800731

[B63] Piao Y. Kim J. Na H. B. Kim D. Baek J. S. Ko M. K. (2008b). Wrap-bake-peel Process for Nanostructural Transformation from β-FeOOH Nanorods to Biocompatible Iron Oxide Nanocapsules. Nat. Mater 7, 242–247. 10.1038/nmat2118 18278051

[B64] Ramanathan S. Oyama S. T. (1995). New Catalysts for Hydroprocessing: Transition Metal Carbides and Nitrides. J. Phys. Chem. 99, 16365–16372. 10.1021/j100044a025

[B65] Sardar K. Deepak F. L. Govindaraj A. Seikh M. M. Rao C. N. R. (2005). InN Nanocrystals, Nanowires, and Nanotubes. Small 1, 91–94. 10.1002/smll.200400011 17193356

[B66] Schmid G. (2010). in Nanoparticles: From Theory to Application, 2nd, Completely Revised andGünter Schmid. Updated Edition (Weinheim: Wiley VCH).

[B67] Schwenzer B. Meier C. Masala O. Seshadri R. DenBaars S. P. Mishra U. K. (2005). Synthesis of Luminescing (In,Ga)N Nanoparticles from an Inorganic Ammonium Fluoride Precursor. J. Mater. Chem. 15, 1891–1895. 10.1039/b418203k

[B68] Seo W. S. Jo H. H. Lee K. Park J. T. (2003). Preparation and Optical Properties of Highly Crystalline, Colloidal, and Size-Controlled Indium Oxide Nanoparticles. Adv. Mater. 15, 795–797. 10.1002/adma.200304568

[B69] Shibata M. Kanetaka H. Furuya M. Yokota K. Ogawa T. Kawashita M. (2021). Cytotoxicity Evaluation of Iron Nitride Nanoparticles for Biomedical Applications. J. Biomed. Mater. Res. 109, 1784–1791. –8. 10.1002/jbm.a.37171 33749145

[B70] Shibata M. Ogawa T. Kawashita M. (2019). Synthesis of Iron Nitride Nanoparticles from Magnetite Nanoparticles of Different Sizes for Application to Magnetic Hyperthermia. Ceramics Int. 45, 23707–23714. 10.1016/j.ceramint.2019.08.086

[B71] Sirvio E. H. Sulonen M. Sundquist H. (1982). Abrasive Wear of Ion-Plated Titanium Nitride Coatings on Plasma-Nitrided Steel Surfaces. Thin Solid Films 96, 93–101. 10.1016/0040-6090(82)90217-6

[B72] Sun S. Zeng H. Robinson D. B. Raoux S. Rice P. M. Wang S. X. (2004). Monodisperse MFe2O4(M = Fe, Co, Mn) Nanoparticles. J. Am. Chem. Soc. 126, 273–279. 10.1021/ja0380852 14709092

[B73] Sun X. Li Y. (2004). Ga2O3 and GaN Semiconductor Hollow Spheres. Angew. Chem. Int. Ed. 43, 3827–3831. 10.1002/anie.200353212 15258947

[B74] Tang J. Fabbri J. Robinson R. D. Zhu Y. Herman I. P. Steigerwald M. L. (2004). Solid-Solution Nanoparticles: Use of a Nonhydrolytic Sol−Gel Synthesis to Prepare HfO2and HfxZr1-xO2Nanocrystals. Chem. Mater. 16, 1336–1342. 10.1021/cm049945w

[B75] Tong W. P. Tao N. R. Wang Z. B. Lu J. Lu K. (2003). Nitriding Iron at Lower Temperatures. Science 299, 686–688. 10.1126/science.1080216 12560546

[B76] Vaidhyanathan B. Rao K. J. (1997). Synthesis of Ti, Ga, and V Nitrides: Microwave-Assisted Carbothermal Reduction and Nitridation†. Chem. Mater. 9, 1196–1200. 10.1021/cm9605835

[B77] Vestal C. R. Zhang Z. J. (2003). Synthesis and Magnetic Characterization of Mn and Co Spinel Ferrite-Silica Nanoparticles with Tunable Magnetic Core. Nano Lett. 3, 1739–1743. 10.1021/nl034816k

[B78] Wang J. Grocholl L. Gillan E. G. (2002). Facile Azidothermal Metathesis Route to Gallium Nitride Nanoparticles. Nano Lett. 2, 899–902. 10.1021/nl0256356

[B79] Wang L. Huo M. Chen Y. Shi J. (2018). Iron-engineered Mesoporous Silica Nanocatalyst with Biodegradable and Catalytic Framework for Tumor-specific Therapy. Biomaterials 163, 1–13. 10.1016/j.biomaterials.2018.02.018 29452944

[B80] Wen J. Yang K. Liu F. Li H. Xu Y. Sun S. (2017). Diverse Gatekeepers for Mesoporous Silica Nanoparticle Based Drug Delivery Systems. Chem. Soc. Rev. 46, 6024–6045. 10.1039/c7cs00219j 28848978

[B81] Xu Z. Xiao F.-S. Purnell S. K. Alexeev O. Kawi S. Deutsch S. E. (1994). Size-dependent Catalytic Activity of Supported Metal Clusters. Nature 372, 346–348. 10.1038/372346a0

[B82] Yamamoto T. A. Nishimaki K. Harabe T. Shiomi K. Nakagawa T. Katsura M. (1999). Magnetic Composites Composed of Iron-Nitride Nanograins Dispersed in a Silver Matrix. Nanostructured Mater. 12, 523–526. 10.1016/S0965-9773(99)00174-9

[B83] Yang C.-T. Huang M. H. (2005). Formation of Arrays of Gallium Nitride Nanorods within Mesoporous Silica SBA-15. J. Phys. Chem. B. 109, 17842–17847. 10.1021/jp052228k 16853288

[B84] Yang G. Phua S. Z. F. Bindra A. K. Zhao Y. (2019). Degradability and Clearance of Inorganic Nanoparticles for Biomedical Applications. Adv. Mater. 31, 1805730. 10.1002/adma.201805730 30614561

[B85] Yi D. K. Lee S. S. Papaefthymiou G. C. Ying J. Y. (2006). Nanoparticle Architectures Templated by SiO2/Fe2O3Nanocomposites. Chem. Mater. 18, 614–619. 10.1021/cm0512979

[B86] Yi D. K. Selvan S. T. Lee S. S. Papaefthymiou G. C. Kundaliya D. Ying J. Y. (2005). Silica-coated Nanocomposites of Magnetic Nanoparticles and Quantum Dots. J. Am. Chem. Soc. 127, 4990–4991. 10.1021/ja0428863 15810812

[B87] Yin M. Gu Y. Kuskovsky I. L. Andelman T. Zhu Y. Neumark G. F. (2004). Zinc Oxide Quantum Rods. J. Am. Chem. Soc. 126, 6206–6207. 10.1021/ja031696+ 15149198

[B88] Yin M. O'Brien S. (2003). Synthesis of Monodisperse Nanocrystals of Manganese Oxides. J. Am. Chem. Soc. 125, 10180–10181. 10.1021/ja0362656 12926934

[B89] Yin M. Wu C.-K. Lou Y. Burda C. Koberstein J. T. Zhu Y. (2005). Copper Oxide Nanocrystals. J. Am. Chem. Soc. 127, 9506–9511. 10.1021/ja050006u 15984877

[B90] Yu F. Zhou H. Zhu Z. Sun J. He R. Bao J. (2017). Three-Dimensional Nanoporous Iron Nitride Film as an Efficient Electrocatalyst for Water Oxidation. ACS Catal. 7, 2052–2057. 10.1021/acscatal.6b03132

[B91] Zettsu N. McLellan J. M. Wiley B. Yin Y. Li Z.-Y. Xia Y. (2006). Synthesis, Stability, and Surface Plasmonic Properties of Rhodium Multipods, and Their Use as Substrates for Surface-Enhanced Raman Scattering. Angew. Chem. 118, 1310–1314. 10.1002/ange.200503174 16416480

[B92] Zhang Y. Ouyang B. Xu J. Jia G. Chen S. Rawat R. S. (2016). Rapid Synthesis of Cobalt Nitride Nanowires: Highly Efficient and Low-Cost Catalysts for Oxygen Evolution. Angew. Chem. 128, 8812–8816. 10.1002/ange.201604372 27254484

[B93] Zhao H. Lei M. Chen X. Tang W. (2006). Facile Route to Metal Nitrides through Melamine and Metal Oxides. J. Mater. Chem. 16, 4407–4412. 10.1039/b611381h

[B94] Zhao H. Lei M. Yang X. a. Jian J. Chen X. (2005). Route to GaN and VN Assisted by Carbothermal Reduction Process. J. Am. Chem. Soc. 127, 15722–15723. 10.1021/ja055877i 16277512

[B95] Zhao W. Chen H. Li Y. Li L. Lang M. Shi J. (2008). Uniform Rattle-type Hollow Magnetic Mesoporous Spheres as Drug Delivery Carriers and Their Sustained-Release Property. Adv. Funct. Mater. 18, 2780–2788. 10.1002/adfm.200701317

