# Supplementary material for: A Facile Route for the Preparation of Monodisperse Iron nitride at Silica Core/shell Nanostructures
Source: Front Bioeng Biotechnol. 2021 Sep 20;9:735727. doi: 10.3389/fbioe.2021.735727 (PMC8488142; doi:10.3389/fbioe.2021.735727)
Supplement: Supplementary file 1 [file Table1.DOCX]

Supplementary Material

Hoonsub Kim^1^, Pyung Won Im^2,3^, Yuanzhe Piao^1,4*^

^1^ Graduate School of Convergence Science and Technology, Seoul National University, Suwon, Republic of Korea

^2^ Department of Neurosurgery Clinical Research Institute Seoul National University Hospital, South Korea

^3^ Cancer Research Institute Ischemia/Hypoxia Disease Institute Seoul National University College of Medicine, Seoul, Republic of Korea

^4^ Advanced Institutes of Convergence Technology, Suwon, Republic of Korea

*** Correspondence:**Yuanzhe Piao
parkat9@snu.ac.kr

# Supplementary Figures


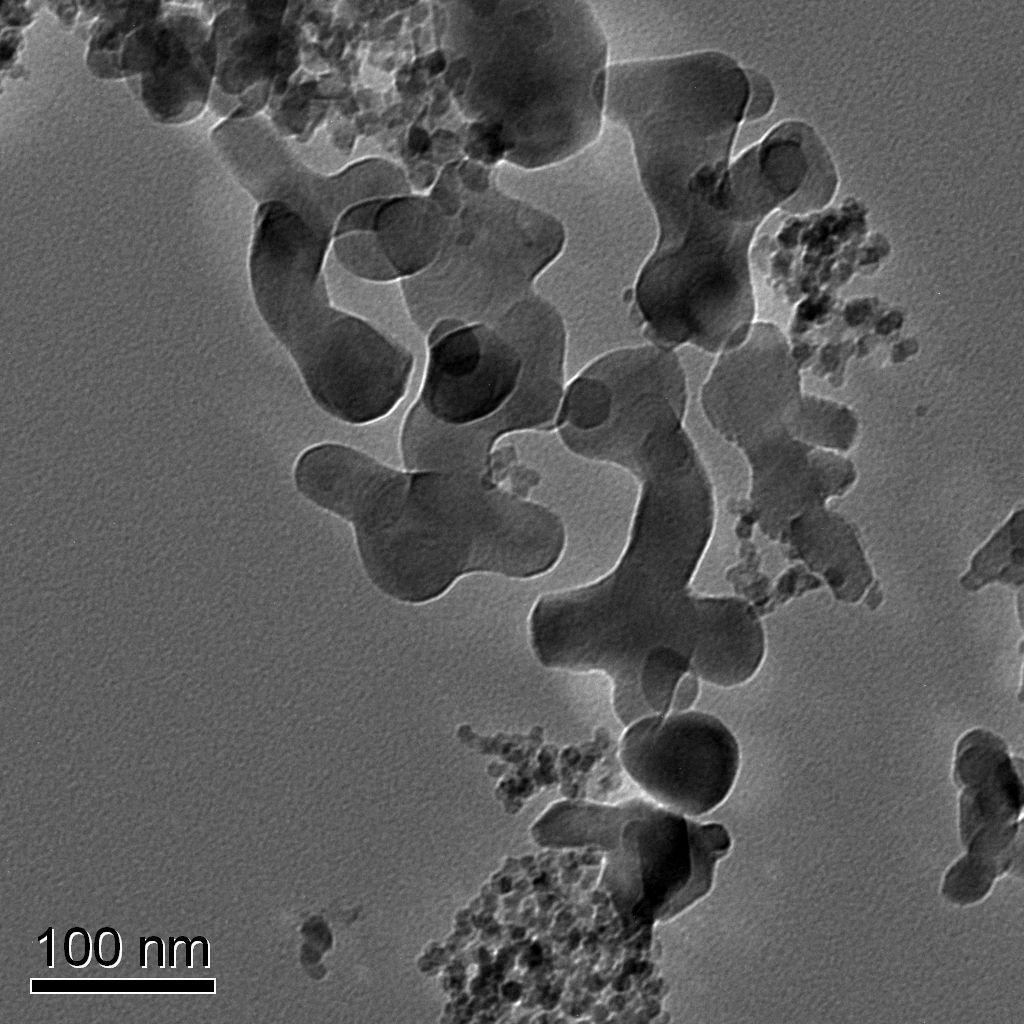


**Supplementary Figure 1.** TEM image of the iron oxide nanoparticles without silica coating after reacted with ammonia gas at 500 °C for 10 h.


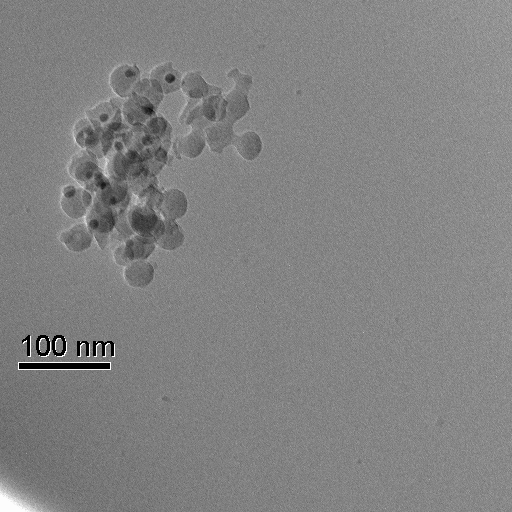


**Supplementary Figure 2.** TEM image of the silica coated iron oxide nanoparticles after reacted with ammonia gas at 700 °C for 10 h.

**
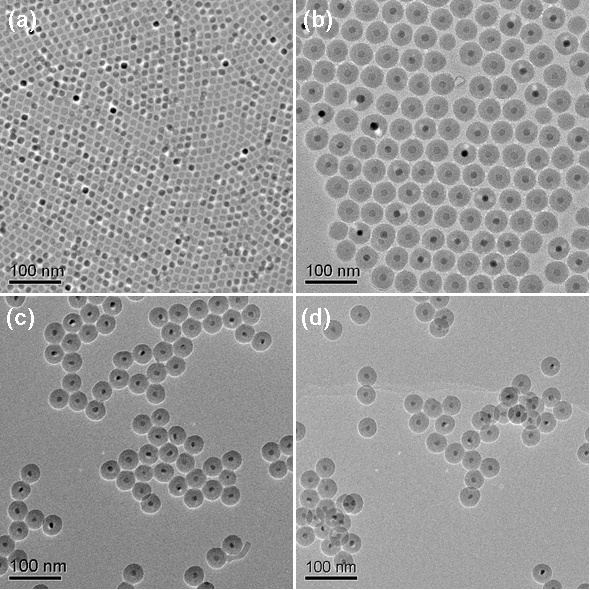
**

**Supplementary Figure 3.** Enlarged TEM images of **(a)** the starting iron oxide nanoparticles of 10.3 nm, **(b)** iron oxide@silica core/shell nanoparticles, and **(c,d)** iron nitride@silica core/shell nanoparticles transferred from **(b)**.


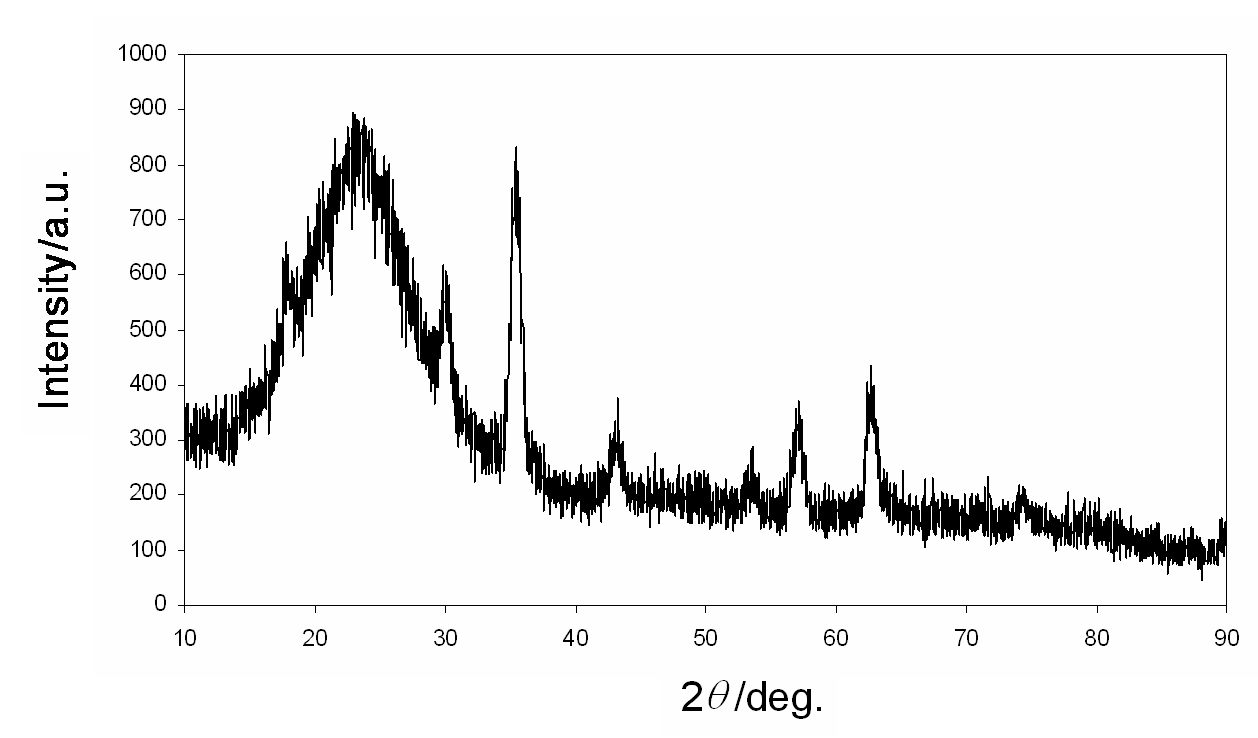


**Supplementary Figure 4.** XRD patterns of 10.3 nm iron oxide nanoparticles coated with silica nanoshells.


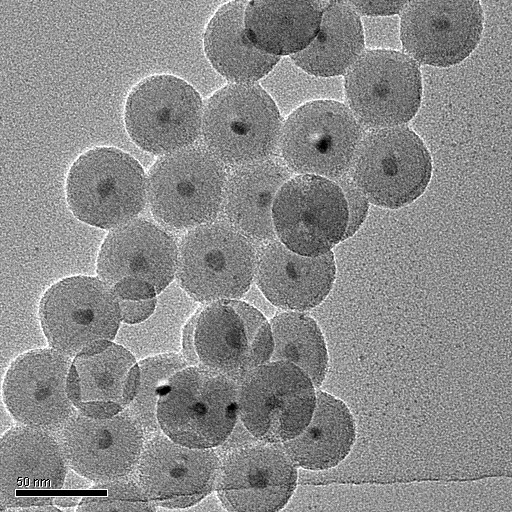


**Supplementary Figure 5.** TEM image of iron nitride@silica core/shell nanoparticles after etching with 2 M H_2_SO_4_ for 10 days.


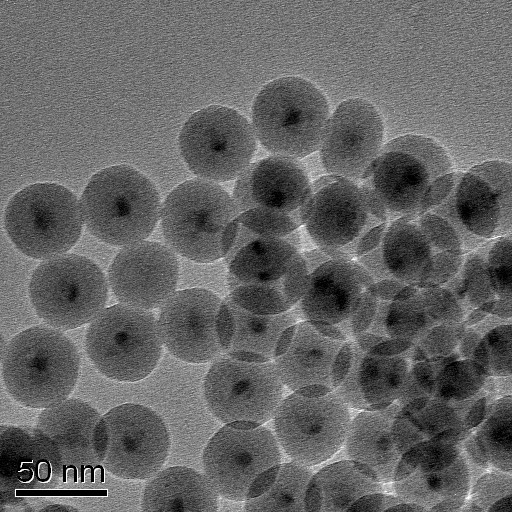


**Supplementary Figure 6.** TEM image of iron nitride@silica core/shell nanoparticles after etching with 2 M HNO_3_ for 10 days.

**Supplementary Figure 7.** Cell viability data of the iron nitride@silica nanostructure material at various concentrations by CCK-8 assay on U87MG, FsaII, and HFB-141103 cell lines for 24 h.
